# Eosinophil is a predictor of severe immune-related adverse events induced by ipilimumab plus nivolumab therapy in patients with renal cell carcinoma: a retrospective multicenter cohort study

**DOI:** 10.3389/fimmu.2024.1483956

**Published:** 2025-01-09

**Authors:** Yoshihiko Tasaki, Shuzo Hamamoto, Shimpei Yamashita, Junya Furukawa, Kazutoshi Fujita, Ryotaro Tomida, Makito Miyake, Noriyuki Ito, Hideto Iwamoto, Yoshihisa Mimura, Yosuke Sugiyama, Rei Unno, Atsushi Okada, Takahiro Yasui, Yoko Furukawa-Hibi

**Affiliations:** ^1^ Department of Clinical Pharmaceutics, Nagoya City University Graduate School of Medical Sciences, Nagoya, Aichi, Japan; ^2^ Department of Nephro-urology, Nagoya City University Graduate School of Medical Sciences, Nagoya, Aichi, Japan; ^3^ Department of Urology, Wakayama Medical University, Wakayama, Japan; ^4^ Department of Urology, Tokushima University Graduate School of Biomedical Sciences, Tokushima, Japan; ^5^ Department of Urology, Kindai University Faculty of Medicine, Osaka, Japan; ^6^ Department of Urology, Nara Medical University, Nara, Japan; ^7^ Department of Urology, Japanese Red Cross Wakayama Medical Center, Wakayama, Japan; ^8^ Division of Urology, Department of Surgery, Tottori University Faculty of Medicine Graduate School of Medicine, Tottori, Japan

**Keywords:** eosinophil, ipilimumab, nivolumab, immune-related adverse event, renal cell carcinoma

## Abstract

**Introduction:**

Immune-related adverse events (irAEs) induced by immune checkpoint inhibitors are difficult to predict and can lead to severe events. Although it is important to develop strategies for the early detection of severe irAEs, there is a lack of evidence on irAEs associated with ipilimumab plus nivolumab therapy for metastatic renal cell carcinoma (RCC). Therefore, this study aimed to investigate the association between eosinophil and severe irAEs in patients receiving ipilimumab plus nivolumab therapy for RCC.

**Methods:**

In this retrospective study, 161 patients receiving ipilimumab plus nivolumab therapy for RCC were divided into three groups based on whether they experienced <grade 2 irAEs (non-severe irAE group), ≥grade 3 irAEs (severe irAE group), or not (non-irAE group). We examined the proportion of eosinophils before and 2 weeks after treatment (baseline and 2-week samples, respectively).

**Results:**

Although the eosinophil in the baseline samples did not differ between the severe irAE and non-irAE groups (2.8% vs. 2.5%, *P* = 0.75), regarding the 2-week samples, the eosinophil was significantly higher in the severe irAE group (mean, 6.6% vs. 3.3%; *P* < 0.05). Multivariate analysis showed that an eosinophil of ≥3.0% was a risk factor for severe irAEs (odds ratio, 6.01). Median progression-free survival (mPFS), mPFS from the start of ipilimumab plus nivolumab therapy to second-line therapy (mPFS2), and median overall survival (mOS) were the shortest in the non-irAE group. Although the mPFS did not differ between the severe and non-severe irAE groups (9.2 vs 14.2 months, *P* = 0.45), notably, mPFS2 and mOS in the former group tended to be shorter than those in the latter group (mPFS2: 29.2 vs not reached, *P* = 0.10; mOS: 36.9 vs 52.3 months, *P* = 0.06).

**Discussion:**

An increased eosinophil 2 weeks after ipilimumab plus nivolumab therapy may be a predictor of severe irAEs, which are associated with poor prognoses, compared with non-severe irAEs among patients with RCC. We provide a novel rationale for the importance of monitoring eosinophil counts for the early detection of severe irAEs.

## 1 Introduction

Immune checkpoint inhibitors (ICIs) have markedly changed the treatment strategies and improved the prognosis of patients with various cancers. Patients with metastatic renal cell carcinoma (RCC) are among those who have benefited from the introduction of ICIs (1–7). A large-scale clinical trial with a follow-up period of approximately 5 years showed that ipilimumab plus nivolumab therapy, which is used as first-line therapy for RCC, improved the median overall survival (mOS) and progression-free survival (mPFS) compared with sunitinib [mOS: 47.0 vs 26.6 months, mPFS: 11.6 vs 8.3 months (1)].

Ipilimumab plus nivolumab therapy causes immune-related adverse events (irAEs) in organs throughout the body. A large-scale clinical trial showed that approximately 90% of patients who received ipilimumab plus nivolumab therapy for RCC developed irAEs of any grade and 46% of these patients developed severe and fatal irAEs (8). The incidence of severe irAEs associated with ipilimumab plus nivolumab therapy is higher than that associated with ICI monotherapy (8, 9). On the other hand, many studies have demonstrated that the occurrence of irAEs is associated with improved clinical outcomes (10–16). However, the association between the severity of irAEs and clinical outcomes in patients treated with ipilimumab plus nivolumab therapy for RCC remains unclear. Additionally, because irAEs are nonspecific immune responses to a wide variety of organs associated with immune activation, owing to the characteristics of these events, it is difficult to predict in advance when and in which organs irAEs will occur. To date, there is no way to detect the early phase of irAEs or prevent severe irAEs, and the only treatment for severe irAEs is to discontinue the treatment and administer immunosuppressants after the occurrence of irAEs. Therefore, it is necessary to reveal the association between the severity of irAEs and clinical outcomes and establish a predictor for the early detection of severe irAEs.

We previously conducted studies focusing on the eosinophil as a predictor of irAEs (15, 16). We found that an increased eosinophil to be a predictor of irAE occurrence in patients with various cancers, including RCC (15, 16). In this study, we investigated the association between the severity of irAEs and clinical outcomes and whether the eosinophil is a predictor of severe irAE occurrence in a larger external cohort.

## 2 Methods

### 2.1 Patient characteristics and study design

**Figure 1:**
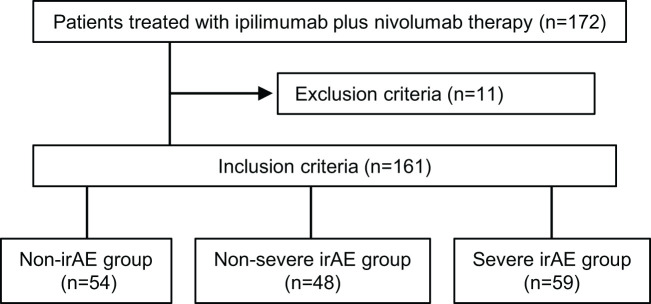
Schema of patient enrollment.

The data of 172 patients treated with ipilimumab plus nivolumab therapy (1 mg/kg ipilimumab and 240 mg/body nivolumab on day 1, every 3 weeks) for RCC between October 2015 and June 2023 at eight hospitals in Japan (Nagoya City University Hospital, Wakayama Medical University Hospital, Kobe University Hospital, Tokushima University Hospital, Kindai University Hospital, Nara Medical University Hospital, Japanese Red Cross Wakayama Medical Center, and Tottori University Hospital) were retrospectively obtained and analyzed. Eleven patients with eosinophilic disorders or those whose eosinophil proportion was not measured before treatment initiation were excluded. Therefore, eventually, 161 patients were evaluated (**Figure 1**). All patients were followed up until death or loss of contact. Eosinophil proportions 1 week before (baseline sample), 2 weeks after one course of treatment (2-week sample), and 3 weeks after one course of treatment (3-week sample) were determined according to our previous studies (15, 16). In addition, to determine the predictors of irAE occurrence, the systemic neutrophil-to-lymphocyte ratio (NLR), platelet-to-lymphocyte ratio (PLR), and C-reactive protein-to-albumin ratio (CAR) were analyzed, as in our previous study (16).

### 2.2 Efficacy data

The best clinical response according to the Response Evaluation Criteria in Solid Tumors (RECIST), version 1.1 (17), including complete response (CR), partial response (PR), stable disease (SD), and progressive disease (PD), was determined. Overall survival (OS) was defined as the period from the start of ipilimumab plus nivolumab therapy to death. Progression-free survival 2 (PFS2) refers to the period from the start of ipilimumab plus nivolumab therapy to second-line therapy.

### 2.3 Safety data

Symptoms that activated the immune system and/or required treatment with immunosuppressants were categorized as irAEs. We divided irAEs into six disease groups, such as endocrine, skin, gastrointestinal, pulmonary, and others (arthritis, demyelinating polyneuropathy, dysgeusia, encephalitis, elevation of serum creatine kinase, fever, hyponatremia, hypokalemia, infusion reaction, myocarditis, myasthenia gravis, myositis, myoclonus, optic neuritis, and renal disorder). Grading of irAEs was based on the National Cancer Institute Common Terminology Criteria for Adverse Events version 5.0. Grade ≥3 irAEs were defined as severe irAEs. The patients were divided into two groups based on whether or not they experienced irAEs (irAE and non-irAE groups, respectively). The irAE group was further subdivided based on irAE severity: Patients who experienced <grade 2 and ≥grade 3 irAEs were categorized into non-severe and severe irAE groups, respectively.

### 2.4 Statistical analyses

A *P*-value of <0.05 was considered statistically significant. Fisher’s exact test was used to assess differences in patient characteristics. Differences in the quantified data between groups were compared using the Welch t-test followed by Bonferroni test in the case of non-homoscedasticity or one-way analysis of variance (ANOVA) followed by Tukey’s test in the case of homoscedasticity. Because the number of patients was sufficient, normality was observed. All reported *P*-values were two-sided. The optimal cutoff values were determined from receiver operating characteristic (ROC) curves. The median OS (mOS), PFS (mPFS), and PFS2 (mPFS2) were calculated using the Kaplan–Meier method and log-rank tests. Statistical analyses were performed using GraphPad Prism 9 software and EZR [Saitama Medical Center, Jichi Medical University, Saitama, Japan (18)].

## 3 Results

### 3.1 Patient characteristics

Patient characteristics are shown in **Table 1**. Among the 161 patients enrolled in the current study, 33.5% (n = 54), 29.8% (n = 48), and 36.7% (n = 59) belonged to the non-irAE, non-severe irAE, and severe irAE groups, respectively. The proportions of patients aged >65 and <65 years were 64.0% (n = 103) and 34.2% (n = 55), respectively. Of the patients, 81.4% (n = 131) were male and 18.6% (n = 30) were female. Regarding International Metastatic RCC Database Consortium (IMDC) risk, 57.1% (n = 92) and 42.9% (n = 69) of the patients were classified into the intermediate and poor IMDC risk groups, respectively. Among the components of IMDC risk classification, diagnosis-to-treatment time of <1 year (73.3%, n = 118) was associated with the highest positivity rate, followed by hemoglobin level < the upper limit of normal (ULN; 62.7%, n = 101), neutrophil count > ULN (27.3%, n = 44), Karnofsky Performance Status score < 80 (22.4%, n = 36), platelet count > ULN (19.3%, n = 31), and calcium level > ULN (9.9%, n = 16). The proportions of patients diagnosed with clear-cell or non-clear-cell carcinoma (papillary, chromophobe, Bellini duct, spindle, and others) were 67.1% (n = 108) and 24.2% (n = 39), respectively. Tissues that were difficult to classify pathologically were diagnosed as RCC after consultation with an experienced urologist and radiologist (8.7%, n = 14). The proportion of patients with sarcomatoid changes was 11.2% (n = 18). The metastasis sites were as follows: bone, 31.7% (n = 51); liver, 14.9% (n = 24); lung, 59.0% (n = 95); and others (abdominal wall, adrenal glands, brain, contralateral kidney, dorsal muscles, gluteal muscle, inferior vena cava, lymph node, pancreas, peritoneal dissemination, subcutaneous tissue, tooth ridge, urethra, iliopsoas muscle, and pleura), 55.3% (n = 89). At the start of ipilimumab plus nivolumab therapy, 1.2% (n = 2) of the patients continuously used steroids. Proportion of patients who treated steroid or steroid pulse therapy due to irAEs was 38.5% (n = 62) and 11.2% (n = 18), respectively. The ratios of IMDC risk classification, Karnofsky Performance Status, histological subtypes, sarcomatoid changes, and proportion of patients who treated steroid or steroid pulse therapy differed significantly among the irAE, non-severe irAE, and severe irAE groups.

**Table 1:** Clinical features of patients between non-irAE, non-severe irAE, and severe irAE groups.

Characteristics, n (%)	Total	Non-irAE group	irAE group		*P* value
			Non-severe irAE group	Severe irAE group	
	161 (100)	54 (33.5)	48 (29.8)	59 (36.7)	
Age, n (%)					0.22
<65 years	55 (34.2)	22 (40.7)	12 (25.0)	21 (35.6)	
≥65 years	103 (64.0)	32 (59.3)	35 (72.9)	36 (61.0)	
Deficit	3 (1.8)	0 (0.0)	1 (2.1)	2 (3.4)	
Gender					0.67
Male	131 (81.4)	46 (85.2)	38 (79.2)	47 (79.7)	
Female	30 (18.6)	8 (14.8)	10 (20.8)	12 (20.3)	
IMDC risk classification					0.10
Intermediate	92 (57.1)	23 (42.6)	32 (66.7)	37 (62.7)	
Poor	69 (42.9)	31 (57.4)	16 (33.3)	22 (37.3)	
Diagnosis-to-treatment time<1 year					0.99
No	43 (26.7)	15 (27.8)	12 (25.0)	16 (27.1)	
Yes	118 (73.3)	39 (72.2)	36 (75.0)	43 (72.9)	
Karnofsky Performance Status					<0.05
≥80	125 (77.6)	34 (63.0)	40 (83.3)	51 (86.4)	
<80	36 (22.4)	20 (37.0)	8 (16.7)	8 (13.6)	
Hemoglobin<ULN					0.19
No	60 (37.3)	15 (27.8)	21 (43.8)	24 (40.7)	
Yes	101 (62.7)	39 (72.2)	27 (56.2)	35 (59.3)	
Calcium>ULN					0.11
No	145 (90.1)	45 (75.9)	44 (91.7)	56 (94.9)	
Yes	16 (9.9)	9 (24.1)	4 (8.3)	3 (5.1)	
Neutrophils>ULN					0.77
No	117 (72.7)	41 (75.9)	35 (72.9)	41 (69.5)	
Yes	44 (27.3)	13 (24.1)	13 (27.1)	18 (30.5)	
Platelets>ULN					0.50
No	130 (80.7)	41 (75.9)	40 (83.3)	49 (83.1)	
Yes	31 (19.3)	13 (24.1)	8 (16.7)	10 (16.9)	
Histological subtype					<0.05
Clear cell	108 (67.1)	30 (55.6)	38 (79.2)	40 (67.8)	
Non-clear cell	39 (24.2)	16 (29.6)	9 (18.8)	14 (23.7)	
Papillary	17	6	4	7	
Chromophobe	4	2	1	1	
Bellini duct carcinoma	3	1	0	2	
Spindle	2	1	0	1	
Others	13	6	4	3	
Unknown	14 (8.7)	8 (14.8)	1 (2.0)	5 (8.5)	
Sarcomatoid change					<0.05
No	143 (88.8)	48 (88.9)	46 (95.8)	49 (83.1)	
Yes	18 (11.2)	6 (11.1)	2 (4.2)	10 (16.9)	
Metastasis site, Bone					0.67
No	110 (68.3)	35 (64.8)	35 (72.9)	40 (67.8)	
Yes	51 (31.7)	19 (35.2)	13 (27.1)	19 (32.2)	
Metastasis site, Liver					0.51
No	137 (85.1)	44 (81.5)	43 (89.6)	50 (84.7)	
Yes	24 (14.9)	10 (18.5)	5 (10.4)	9 (15.3)	
Metastasis site, Lung					0.62
No	66 (41.0)	20 (37.0)	19 (39.6)	27 (45.8)	
Yes	95 (59.0)	34 (63.0)	29 (60.4)	32 (54.2)	
Metastasis site, Others					0.75
No	72 (44.7)	22 (40.7)	23 (47.9)	27 (45.8)	
Yes	89 (55.3)	32 (59.3)	25 (52.1)	32 (54.2)	
Steroids before ipilimumab plus nivolumab					0.64
Use of steroids	2 (1.2)	1 (1.9)	0 (0.0)	1 (1.7)	
No use of steroids	159 (98.8)	53 (98.1)	48 (100)	58 (98.3)	
Patients who treated steroids due to irAEs					<0.05
No	99 (61.5)	54 (100)	31 (64.6)	14 (23.7)	
Yes	62 (38.5)	0 (0.0)	17 (35.4)	45 (76.3)	
Patients who treated steroid pulse therapy due to irAEs					<0.05
No	143 (88.8)	54 (100)	48 (100)	41 (69.5)	
Yes	18 (11.2)	0 (0.0)	0 (0.0)	18 (30.5)	

IMDC, International Metastatic Renal Cell Carcinoma Database Consortium; irAEs, immune-related adverse events; ULM, upper limit of normal.

### 3.2 Therapeutic features

**Table 2** shows the therapeutic history of the patients enrolled in this study. Regarding the number of courses of ipilimumab plus nivolumab therapy, 12.4% (n = 20), 13.7% (n = 22), 16.1% (n = 26), and 57.8% (n = 93) of the patients received 1, 2, 3, and 4 courses, respectively. The percentages of patients who underwent nephrectomy before or after ipilimumab plus nivolumab therapy were 52.8% (n = 85) and 5.6% (n = 9), respectively. Nivolumab monotherapy after ipilimumab plus nivolumab therapy was administered to 60.2% (n = 97) of the patients. The median number of courses of nivolumab monotherapy was 8 (range: 1-66). Regarding additional treatment, 49.1% (n = 79) of the patients received second-line therapy, such as axitinib (19.3%, n = 31), cabozantinib (14.3%, n = 23), pazopanib (8.7%, n = 14), sunitinib (4.3%, n = 7), temsirolimus (1.2%, n = 2), and sorafenib (0.6%, n = 1) therapies. The percentage of patients who received third-line therapy was 20.5% (n = 33). Consistent with the numbers associated with second-line therapy, the proportion of patients who received axitinib (8.0%, n = 13) as third-line therapy was the highest, followed by those of patients who received cabozantinib (6.2%, n = 10), pazopanib (3.1%, n = 5), sunitinib (1.2%, n = 2), everolimus (0.6%, n = 1), nivolumab monotherapy (0.6%, n = 1), and sorafenib (0.6%, n = 1). The proportions of patients who received fourth- and fifth-line therapies were 5.6% (n = 9) and 1.8% (n = 3), respectively. The percentages of treatment courses, nephrectomy before or after ipilimumab plus nivolumab therapy, and nivolumab monotherapy after ipilimumab plus nivolumab therapy significantly differed among the irAE, non-severe irAE, and severe irAE groups.

**Table 2:** Therapeutic features.

Characteristics	Total	Non-irAE group	irAE group		*P* value
			Non-severe irAE group	Severe irAE group	
n (%)	161 (100)	54 (33.5)	48 (29.8)	59 (36.7)	
Number of courses of ipilimumab plus nivolumab, n (%)					<0.05
1	20 (12.4)	11 (20.4)	1 (2.0)	8 (13.6)	
2	22 (13.7)	6 (11.1)	5 (10.4)	11 (18.6)	
3	26 (16.1)	5 (9.3)	5 (10.4)	16 (27.1)	
4	93 (57.8)	32 (59.2)	37 (77.1)	24 (40.7)	
Nephrectomy before ipilimumab plus nivolumab, n (%)					<0.05
No	76 (47.2)	35 (64.8)	18 (37.5)	23 (39.0)	
Yes	85 (52.8)	19 (35.2)	30 (62.5)	36 (61.0)	
Nephrectomy after ipilimumab plus nivolumab, n (%)					<0.05
No	152 (94.4)	52 (96.3)	42 (77.5)	58 (98.3)	
Yes	9 (5.6)	2 (3.7)	6 (12.5)	1 (1.7)	
Nivolumab monotherapy after ipilimumab plus nivolumab					<0.05
No	64 (39.8)	25 (46.3)	10 (20.8)	29 (49.2)	
Yes	97 (60.2)	29 (53.7)	38 (79.2)	30 (50.8)	
The median course of nivolumab monotherapy after ipilimumab plus nivolumab, (range)	8 (1-66)	8 (1-62)	9.5 (1-66)	5 (1-51)	0.29
Second-line therapy after ipilimumab plus nivolumab					0.68
No	82 (50.9)	29 (53.7)	25 (52.1)	28 (47.5)	
Yes	79 (49.1)	25 (46.3)	23 (47.9)	31 (52.5)	
Axitinib	31 (19.3)	6	12	13	
Cabozantinib	23 (14.3)	6	6	11	
Pazopanib	14 (8.7)	8	5	1	
Sorafenib	1 (0.6)	1	0	0	
Sunitinib	7 (4.3)	2	0	5	
Temsirolimus	2 (1.2)	1	0	1	
Third-line therapy after ipilimumab plus nivolumab					0.25
No	128 (79.5)	46 (85.2)	34 (70.8)	48 (81.4)	
Yes	33 (20.5)	8 (14.8)	14 (29.2)	11 (18.6)	
Axitinib	13 (8.0)	3	6	4	
Cabozantinib	10 (6.2)	2	4	4	
Everolimus	1 (0.6)	1	0	0	
Nivolumab monotherapy	1 (0.6)	0	1	0	
Pazopanib	5 (3.1)	1	2	2	
Sorafenib	1 (0.6)	1	0	0	
Sunitinib	2 (1.2)	0	1	1	
Fourth-line therapy after ipilimumab plus nivolumab					0.88
No	152 (94.4)	51 (94.4)	45 (93.7)	56 (94.9)	
Yes	9 (5.6)	3 (5.6)	3 (6.3)	3 (5.1)	
Cabozantinib	3 (1.8)	1	1	1	
Nivolumab monotherapy	2 (1.2)	0	0	2	
Pazopanib	2 (1.2)	2	0	0	
Sunitinib	2 (1.2)	0	2	0	
Fifth-line therapy after ipilimumab plus nivolumab					0.98
No	158 (98.2)	53 (98.1)	47 (97.9)	58 (98.3)	
Yes	3 (1.8)	1 (1.9)	1 (2.1)	1 (1.7)	
Axitinib	2 (1.2)	0	1	1	
Cabozantinib	1 (0.6)	1	0	0	

### 3.3 Efficacy and safety profiles

The mOS and mPFS in the current study were 32.6 and 8.4 months, respectively (**Supplementary Figures 1A, B**). Regarding responses to ipilimumab plus nivolumab therapy, complete response, partial response, stable disease, and progressive disease were achieved in 10.5% (n = 17), 34.2% (n = 55), 26.1% (n = 42), and 23.0 (n = 37) of the patients (**Table 3**). The overall response rate (ORR) and disease control rate (DCR) were 44.7% (n = 72) and 70.8% (n = 114), respectively (**Table 3**). The ORR and DCR were significantly higher in the non-severe and severe irAE groups than in the non-irAE group (ORR: 50.0% vs. 57.6% vs. 25.9%; DCR: 77.1% vs. 81.4% vs. 53.7%; *P* < 0.05; **Table 3**).

**Table 3:** Profile of efficacy in ipilimumab plus nivolumab.

Characteristics	Total	Non-irAE group	irAE group		*P* value
			Non-severe irAE group	Severe irAE group	
Total, n (%)	161 (100)	54 (33.5)	48 (29.8)	59 (36.7)	
Best response to ipilimumab plus nivolumab, n (%)					<0.05
Complete response	17 (10.5)	7 (13.0)	3 (6.2)	7 (11.9)	
Partial response	55 (34.2)	7 (13.0)	21 (43.8)	27 (45.8)	
Stable disease	42 (26.1)	15 (27.7)	13 (27.1)	14 (23.7)	
Progression disease	37 (23.0)	21 (38.9)	8 (16.7)	8 (13.5)	
Not evaluable	10 (6.2)	4 (7.4)	3 (6.2)	3 (5.1)	
Overall response rate (ORR), n (%)					<0.05
No	79 (49.1)	36 (66.7)	21 (43.8)	22 (37.3)	
Yes	72 (44.7)	14 (25.9)	24 (50.0)	34 (57.6)	
Not evaluable	10 (6.2)	4 (7.4)	3 (6.2)	3 (5.1)	
Disease control rate (DCR), n (%)					<0.05
No	37 (23.0)	21 (38.9)	8 (16.7)	8 (13.5)	
Yes	114 (70.8)	29 (53.7)	37 (77.1)	48 (81.4)	
Not evaluable	10 (6.2)	4 (7.4)	3 (6.2)	3 (5.1)	

Overall, 107 patients experienced a total of 174 irAEs. Among the 174 events, 57.5% (100 events) and 42.5% (74 events) were grade <2 and grade ≥3 irAEs, respectively (**Supplementary Table 1**). The rate of endocrinal irAEs was the highest (29.9%, 52 events), followed by skin-related (19.5%, 34 events), gastrointestinal (19.5%, 34 events), others (19.0%, 33 events), and pulmonary (12.1%, 21 events) irAEs (**Supplementary Table 1**). The proportions of patients who experienced one and two or more irAEs was 59.8% (n = 64) and 40.2% (n = 43; **Supplementary Table 2**), respectively. The percentage of patients who experienced two or more irAEs was significantly higher in the severe irAE group than in the non-severe irAE group (52.5% vs. 25.0%, *P* < 0.05; **Supplementary Table 2**). The reasons for discontinuing ipilimumab plus nivolumab therapy were irAEs in 39.3% (n = 42) and non-irAEs (disease progression, death, complete remission, and others) in 38.3% (n = 41) of the patients (**Supplementary Table 3**). The proportions of patients who discontinued treatment because of irAEs were not significantly different between the non-severe and severe irAE groups (27.1% vs. 49.2%, *P* = 0.17; **Supplementary Table 3**). The subgroup analysis showed discontinuation due to irAEs did not affect overall survival (**Supplementary Figure 2**).

### 3.4 Analysis of the association between an increased eosinophil and irAE severity

**Figure 2:**
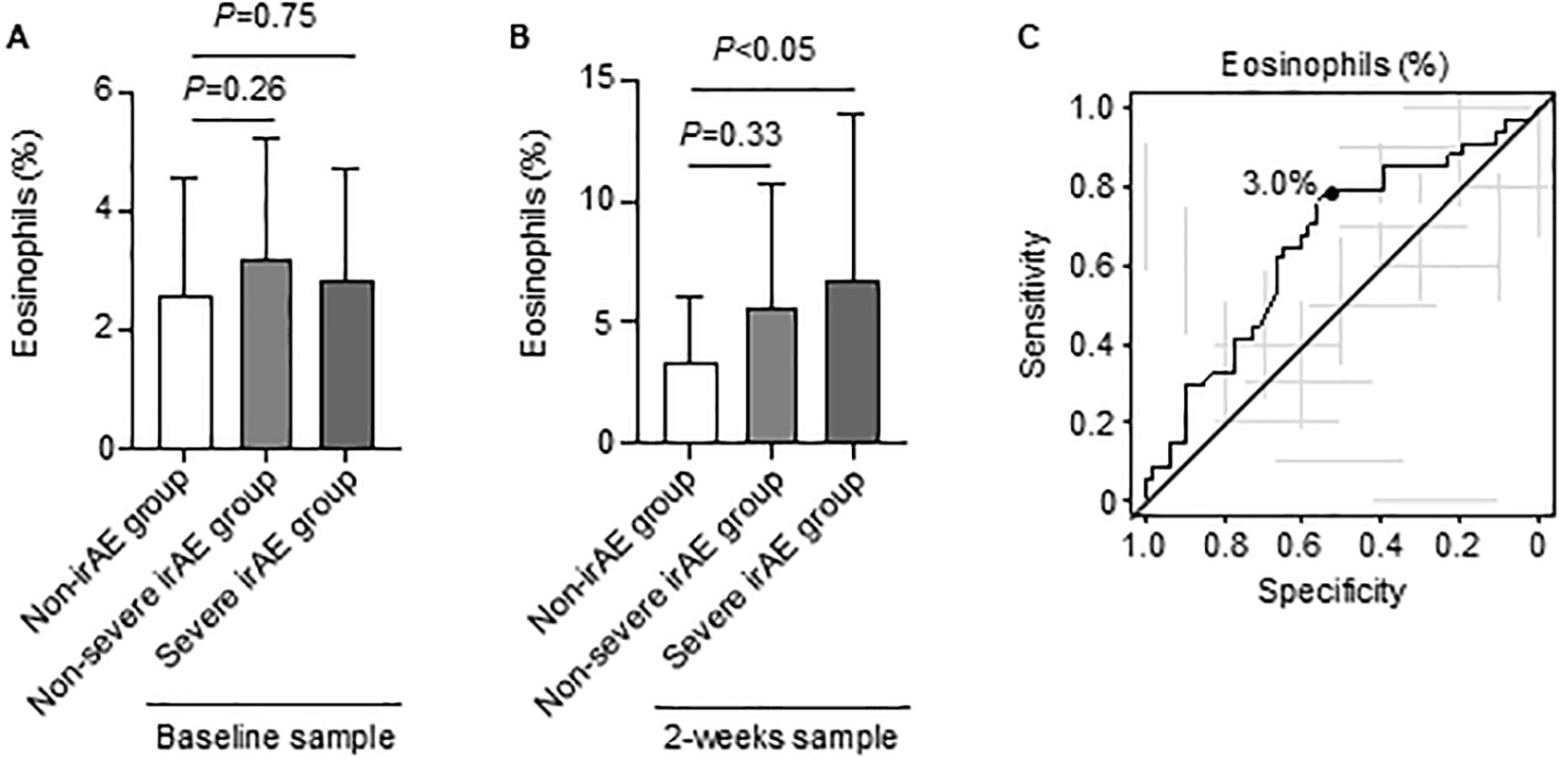
Changes in eosinophils over time. **(A)**, Bar graph showing eosinophils in the baseline samples: non-irAEs (n = 54), non-severe irAEs (n = 48), and severe irAEs (n = 59). **(B)**, Bar graph showing eosinophils in 2-week samples from the non-irAE (n = 28), non-severe irAE (n = 20), and severe irAE (n = 35) groups. **(C)**, Receiver operating characteristic curve analysis of eosinophil count for the occurrence of severe irAEs. Results are presented as the mean ± SD. **(A)** One-way analysis of variance followed by Tukey’s test. **(B)** Welch t-test followed by Bonferroni test. irAEs, immune-related adverse events.

We investigated whether an increased eosinophil was related to irAE severity. No differences in eosinophil were observed between baseline samples (non-irAE: 2.5% vs. non-severe irAE: 3.1%, *P* = 0.26; non-irAE: 2.5% vs. non-severe irAE: 2.8%, *P* = 0.75; **Figure 2A**). Notably, while the eosinophil in the 2-week samples did not differ between the non-irAE and non-severe irAE groups (non-irAE: 3.3% vs. non-severe irAE: 5.5%, *P* = 0.33), and the eosinophil in the severe irAE group was significantly higher than that in the non-irAE group (non-irAE: 3.3% vs. severe irAE: 6.6%, *P* < 0.05; **Figure 2B**). Consistent with that in the 2-week samples, in the 3-week samples, the eosinophil in the severe irAE group tended to be higher than that in the non-irAE group (non-irAE group: 5.1% vs. severe irAE group: 7.1%, *P* = 0.06; **Supplementary Figure 3**). The NLR, PLR, and CAR in baseline samples did not differ among the three groups (**Supplementary Figure 4**).

The optimal cut-off value of the eosinophil proportion in 2-week samples against the occurrence of severe irAEs was 3.0% (area under the curve = 0.653, 95% CI = 0.53–0.77, sensitivity = 0.79, specificity = 0.52; **Figure 2C**). Notably, univariate and multivariate logistic regression analyses showed that an eosinophil proportion of ≥3.0% in the 2-week sample increased the risk for occurrence of severe irAEs (univariate analysis: OR = 4.19, 95% CI = 1.53–11.5; *P* < 0.05; multivariate analysis: OR = 6.01, 95% CI = 1.81–20.0; *P* < 0.05; **Table 4**).

**Table 4:** Univariate and multivariate logistic regression analysis of risk factors for the occurrence of severe irAEs.

	Univariate			Multivariate		
	OR	95%CI	*P* value	OR	95%CI	*P* value
Age: ≥65 years	0.87	0.44-1.71	0.68	0.97	0.34-2.79	0.96
Gender: male	0.83	0.37-1.89	0.67	1.35	0.36-4.92	0.65
IMDC risk group: Poor	0.69	0.36-1.34	0.27	1.93	0.48-7.68	0.35
Karnofsky Performance Status: ≥80	1.95	0.87-4.35	0.10	2.21	0.57-8.54	0.25
Histological subtype, Clear cell: Yes	1.05	0.53-2.09	0.88	1.37	0.45-4.12	0.57
NLR in baseline sample: ≥3.0	1.06	0.55-2.04	0.85	1.10	0.35-3.39	0.87
NLR in 3-week sample: ≥3.0	0.82	0.40-1.66	0.58			
PLR in baseline sample: ≥150	0.95	0.45-2.00	0.89	1.07	0.32-3.52	0.90
PLR in 3-week sample: ≥150	0.50	0.24-1.04	0.06			
Proportion of eosinophils: ≥3.0%	4.19	1.53-11.5	<0.05	6.01	1.81-20.0	<0.05

CI, confidence interval; IMDC, International Metastatic Renal Cell Carcinoma Database Consortium; irAE, immune-related adverse event; OR, odds ratio; NLR, neutrophil-to-lymphocyte ratio; PLR, platelet-to-lymphocyte ratio.

### 3.5 Severe irAEs occurrence associated with poor prognosis

**Figure 3:**
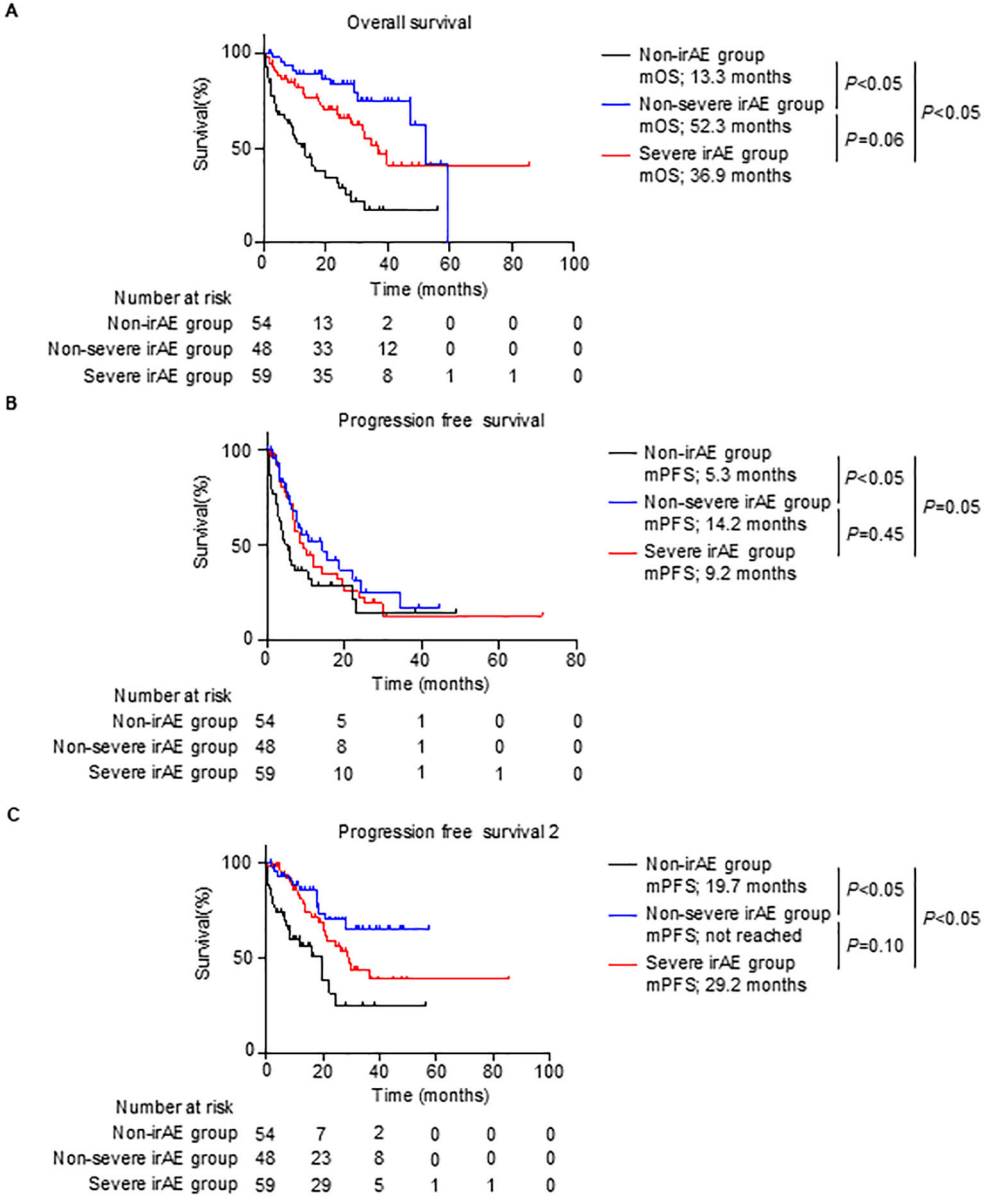
Survival outcomes by grades of irAEs. **(A–C)**, Kaplan–Meier survival curves for **(A)** overall survival rate (non-irAE group, n = 54; non-severe irAE group, n = 48; and severe irAE group, n = 59), **(B)** progression-free survival (non-irAE group, n = 54; non-severe irAE group, n = 48; and severe irAE group, n = 59), and **(C)** progression-free survival 2 (non-irAE group, n = 54; non-severe irAE group, n = 48; and severe irAE group, n = 59) in patients. **(A–C)** Log-rank test. irAE, immune-related adverse events; mOS, median overall survival; mPFS, median progression-free survival.

The mOS and mPFS of the non-irAE group were associated with poor survival compared with those of the non-severe and severe irAE groups (mOS: 13.3 months (non-irAE) vs 52.3 months (non-severe irAE) vs 36.9 months (severe irAE); mPFS: 5.3 months (non-irAE) vs 14.2 months (non-severe irAE) vs 9.2 months (severe irAE); **Figures 3A, B**). Kaplan–Meier analysis showed no statistically significant difference in the mPFS between the non-severe and severe irAE groups (non-severe irAE: 14.2 months, severe irAE: 9.2 months, *P* = 0.45; **Figure 3B**), the mOS tended to be shorter for the severe irAE group than for the non-severe irAE group (non-severe irAE: 52.3 months, severe irAE: 36.9 months, *P* = 0.06; **Figure 3A**).

The differences in the mOS and mPFS between the non-severe and severe irAE groups inspired us to investigate PFS2, which is the PFS until the second-line therapy after ipilimumab plus nivolumab therapy. Consistent with results obtained for mOS and mPFS, the mPFS2 of the non-irAE group was significantly shorter than that of the non-severe and severe irAE groups (non-irAE, 19.7 vs non-severe irAE, not reached vs severe irAE, 29.2 months, *P* < 0.05; **Figure 3C**). Notably, the mPFS2 of the severe irAE group tended to be shorter than that of the non-severe irAE group (non-severe irAE, not reached vs severe irAE, 29.2 months, *P* = 0.10; **Figure 3C**). These results indicated that severe irAE occurrence was associated with a poor prognosis.

## 4 Discussion

In the current study, we identified that an increased eosinophil count in patients treated with ipilimumab plus nivolumab therapy for RCC may be a predictor of severe irAE occurrence, which is associated with poor prognoses, by using a larger external cohort, consistent with our previous studies (15, 16). Notably, to our knowledge, we are the first to report that an increased eosinophil count may be a strong predictor of severe irAE occurrence such as grade ≥3 irAEs in these patients.

Consistent with our data (**Figures 3A, B**), several studies have reported that irAEs induced by ICIs are associated with better clinical outcomes compared with the outcomes in patients who do not have irAEs, including patients having RCC treated with ipilimumab plus nivolumab therapy (10–16). In contrast, as shown in **Figures 3A–C** of the current study, while mPFS was not significantly different between the severe and non-severe irAE groups (9.2 vs 14.2 months, *P* = 0.45), mOS and mPFS2 in the severe irAE group were associated with a poor prognosis compared with those in the non-severe irAE group. These data suggest that the occurrence of severe irAEs negatively affects the long-term prognosis of patients having RCC treated with ipilimumab plus nivolumab therapy. Although the association between irAE severity and clinical outcome in various cancer remains to be fully elucidated, especially in cases of patients treated with ipilimumab plus nivolumab therapy for RCC, Mathieu Grangeon et al. showed that severe irAE occurrence is associated with poor survival compared with non-severe irAE occurrence in patients treated with atezolizumab for non-small cell lung cancer (19). Our findings on the association between irAE severity and prognosis are supported by those of other studies (19). Although a larger data analysis is needed, our data suggest that irAEs should be maintained at a low grade to improve prognosis.

Because irAEs are caused by the activation of immune cells, patients with severe irAEs could theoretically have an immune response that is more responsive to ICIs. Thus, patients who have experienced severe irAEs may have better clinical outcomes (20, 21). In practice, our data showed that although the percentage of patients in the non-severe irAE group who received four courses of ipilimumab plus nivolumab therapy was higher than that in the severe irAE group (**Table 2**), mPFS did not differ between the groups (non-severe irAE: 14.2 vs severe irAE: 9.2 months, *P* = 0.45; **Figure 3B**). Moreover, the ORR and DCR of the severe irAE group were comparable to those of the non-severe irAE group (ORR: non-severe, 50.0%; severe, 57.6%; DCR: non-severe, 77.1%; severe, 81.4%; **Table 1**). In contrast, the proportion of patients who received nephrectomy and nivolumab monotherapy after ipilimumab plus nivolumab therapy was higher in the non-severe irAE group than in the severe irAE group (nephrectomy: 12.5% and 1.7%, nivolumab monotherapy: 79.2% and 50.8%, respectively; **Table 2**). These data suggest that severe irAE occurrence is not associated with a better clinical response and affects long-term survival, including treatment strategies after ipilimumab plus nivolumab therapy, rather than the response to ipilimumab plus nivolumab therapy. Additionally, proportion of patients who treated steroid or steroid pulse therapy due to irAEs was higher in the severe irAE group than in the non-severe irAE group (steroid therapy: 76.3% and 35.4%, steroid pulse therapy: 30.5% and 0.0%, respectively; **Table 1**). Although the impact of steroid or steroid pulse therapy due to irAEs on effect of ICIs is still unclear, high-dose steroid therapy may also have a negative impact on long-term survival.

Because severe irAEs may be associated with poor clinical survival, physicians need to perform early detection and intervention for irAE occurrence to prevent severe irAEs. Therefore, it is necessary to establish predictors. There are several reports on predictors of severe irAEs, such as cytokines, autoantibodies, and blood parameters (22–26). Moreover, several studies reported that the occurrence of irAEs is related to the microbiome or genetic variants (27–31). However, there is a lack of evidence regarding the predictors of severe irAEs in patients treated with ipilimumab plus nivolumab therapy for RCC. To date, no practical or established predictor exists in this context. In the current study, an eosinophil of ≥3.0% was found to increase the risk of developing a severe irAE by six-fold (**Table 4**). We found that an eosinophil of ≥3.0% in 2 weeks after one course of treatment may be a predictor for irAE occurrence, especially for severe irAE occurrence. Therefore, physicians should carefully evaluate changes in eosinophil counts over time to prevent severe irAEs.

Eosinophils are immune cells that attack parasites, bacteria, and viruses and are involved in the development of allergic asthma, esophagitis, myopathies, and autoimmune disorders (32). Recently, some studies have revealed that eosinophils are involved in enhancing antitumor effects by regulating CD8+ T-cell activation in ICI-treated patients with cancer (33). Therefore, many studies have focused on the eosinophil count as a predictor of prognosis and irAE occurrence in patients with various cancers treated using ICIs (15, 16, 34–45). The current study demonstrated that an increased eosinophil count is related to the occurrence of irAEs, especially severe events. Previous findings support our data, in that an increased eosinophil count may reflect safety in RCC treated with ipilimumab plus nivolumab therapy.

This study has methodological limitations. Specifically, we could not control for patient selection bias because this was a retrospective study. Additionally, there were many missing eosinophil data 2 weeks after treatment. Therefore, we plan to confirm our findings in a prospective study.

In conclusion, the development of irAEs is expected to improve prognosis compared with an absence of irAEs; however, severe irAEs may result in a worse prognosis compared with non-severe irAEs. Increased eosinophil may be a predictor of severe irAEs in patients treated with ipilimumab plus nivolumab therapy for RCC. We provide a new strategy for the prediction of irAEs for early detection and prevention of severe irAEs.

## Data Availability

The raw data supporting the conclusions of this article will be made available by the authors, without undue reservation.
