# Supplementary material for: Eosinophil is a predictor of severe immune-related adverse events induced by ipilimumab plus nivolumab therapy in patients with renal cell carcinoma: a retrospective multicenter cohort study
Source: Front Immunol. 2025 Jan 9;15:1483956. doi: 10.3389/fimmu.2024.1483956 (PMC11754295; doi:10.3389/fimmu.2024.1483956)
Supplement: Supplementary file 1 [file DataSheet1.docx]

**Supplementary Table 1. Profile of** **irAEs**

|  | Total events | Grade <2 (%) | Grade ≥3 (%) |
| --- | --- | --- | --- |
| Profile of irAEs, event (%) | 174 (100) | 100 (57.5) | 74 (42.5) |
| Endocrine | 52 (29.9) | 30 | 22 |
| Skin | 34 (19.5) | 29 | 5 |
| Gastrointestinal | 34 (19.5) | 18 | 16 |
| Pulmonary | 21 (12.1) | 6 | 15 |
| Others | 33 (19.0) | 17 | 16 |

irAEs: immune-related adverse events

**Supplementary Table 2. The number of patients who experienced** **multiple irAEs**

| Characteristics | Total | irAE group | | *P* value |
| --- | --- | --- | --- | --- |
|  |  | Non-severe  irAE group | Severe  irAE group |  |
| Total | 107 | 48 | 59 |  |
| Number of patients who experienced one irAEs, n (%) | 64 (59.8) | 36 (75.0) | 28 (47.5) | <0.05 |
| Number of patients who experienced 2 or more irAEs, n (%) | 43 (40.2) | 12 (25.0) | 31 (52.5) | <0.05 |

irAEs: immune-related adverse events

**Supplementary Table 3. Reasons for discontinuing treatment**

| Characteristics | Total | irAE group | | *P* value |
| --- | --- | --- | --- | --- |
|  |  | Non-severe  irAE group | Severe  irAE group |  |
| Total | 107 | 48 | 59 |  |
| Number of patients who discontinued due to irAEs, n (%) |  |  |  | 0.17 |
| No | 65 (60.7) | 35 (72.9) | 30 (50.8) |  |
| Yes | 42 (39.3) | 13 (27.1) | 29 (49.2) |  |
| Number of patients who discontinued due to non irAEs, n (%) |  |  |  | 0.17 |
| No | 66 (61.7) | 29 (60.4) | 37 (62.7) |  |
| Yes | 41 (38.3) | 19 (39.6) | 22 (37.3) |  |

irAEs: immune-related adverse events
