# Supplementary material for: Eosinophil is a predictor of severe immune-related adverse events induced by ipilimumab plus nivolumab therapy in patients with renal cell carcinoma: a retrospective multicenter cohort study
Source: Front Immunol. 2025 Jan 9;15:1483956. doi: 10.3389/fimmu.2024.1483956 (PMC11754295; doi:10.3389/fimmu.2024.1483956)
Supplement: Supplementary file 2 [file Presentation1.pptx]

## Slide 1
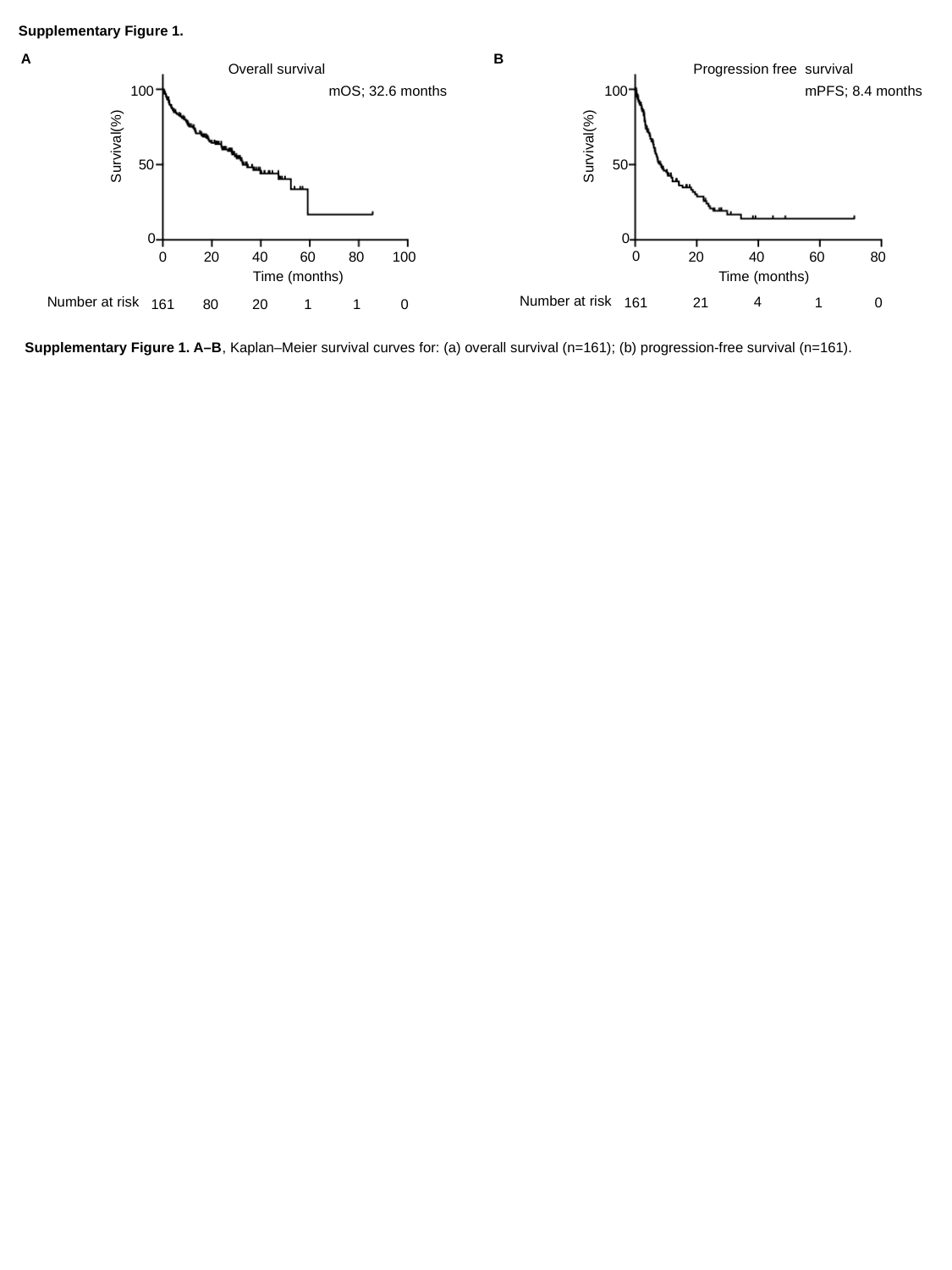

Supplementary Figure 1.
A
B
Progression free survival
Overall survival
mOS; 32.6 months
mPFS; 8.4 months
100
100
Survival(%)
Survival(%)
50
50
0
0
0
0
20
40
60
80
100
20
40
60
80
 Time (months)
 Time (months)
Number at risk
Number at risk
4
161
21
1
0
161
80
20
1
1
0
Supplementary Figure 1. A–B, Kaplan–Meier survival curves for: (a) overall survival (n=161); (b) progression-free survival (n=161).

## Slide 2
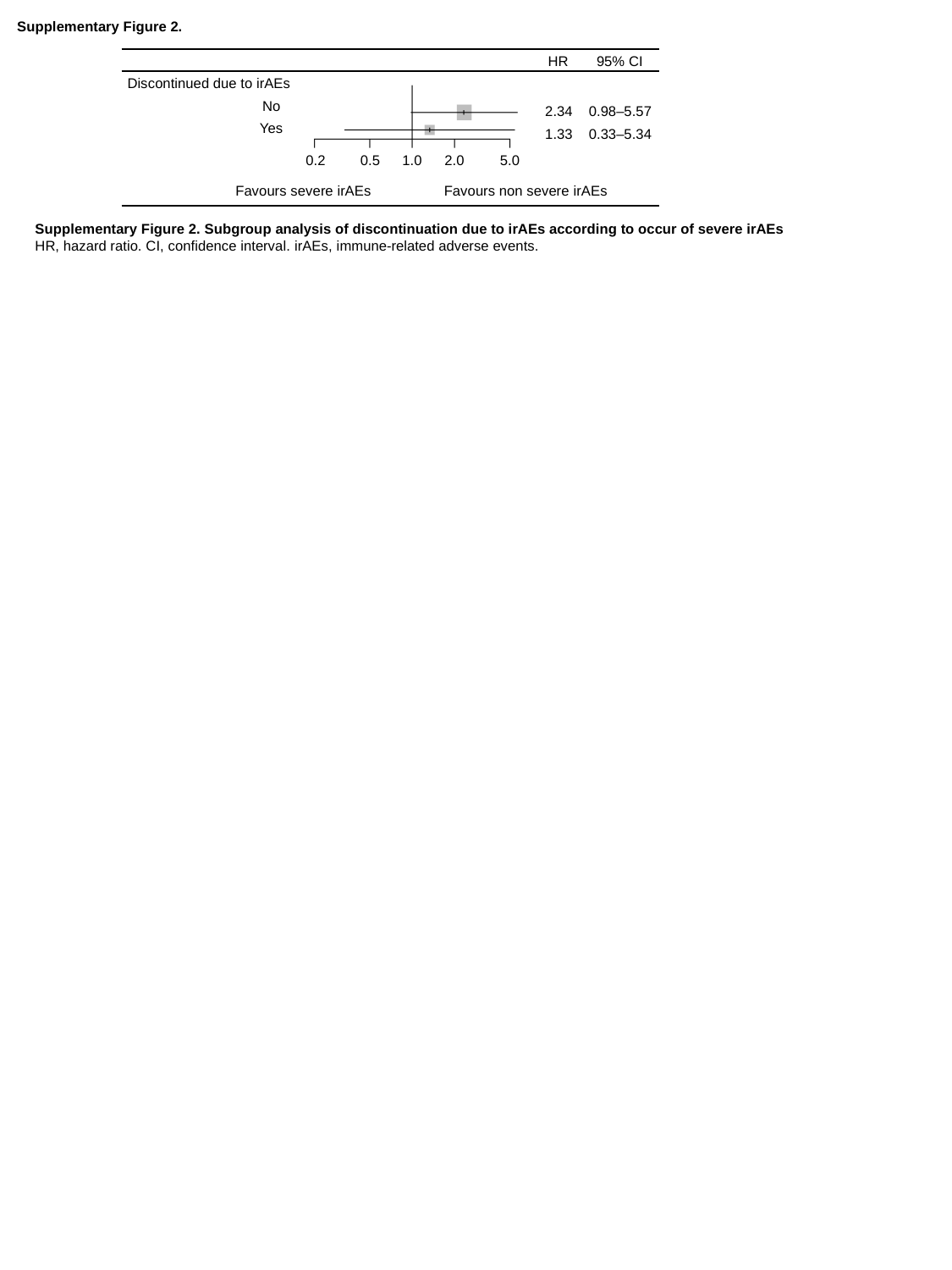

Supplementary Figure 2.
HR
95% CI
 Discontinued due to irAEs
No
2.34
0.98–5.57
Yes
1.33
0.33–5.34
0.2
0.5
1.0
2.0
5.0
Favours severe irAEs
Favours non severe irAEs
Supplementary Figure 2. Subgroup analysis of discontinuation due to irAEs according to occur of severe irAEs
HR, hazard ratio. CI, confidence interval. irAEs, immune-related adverse events.

## Slide 3
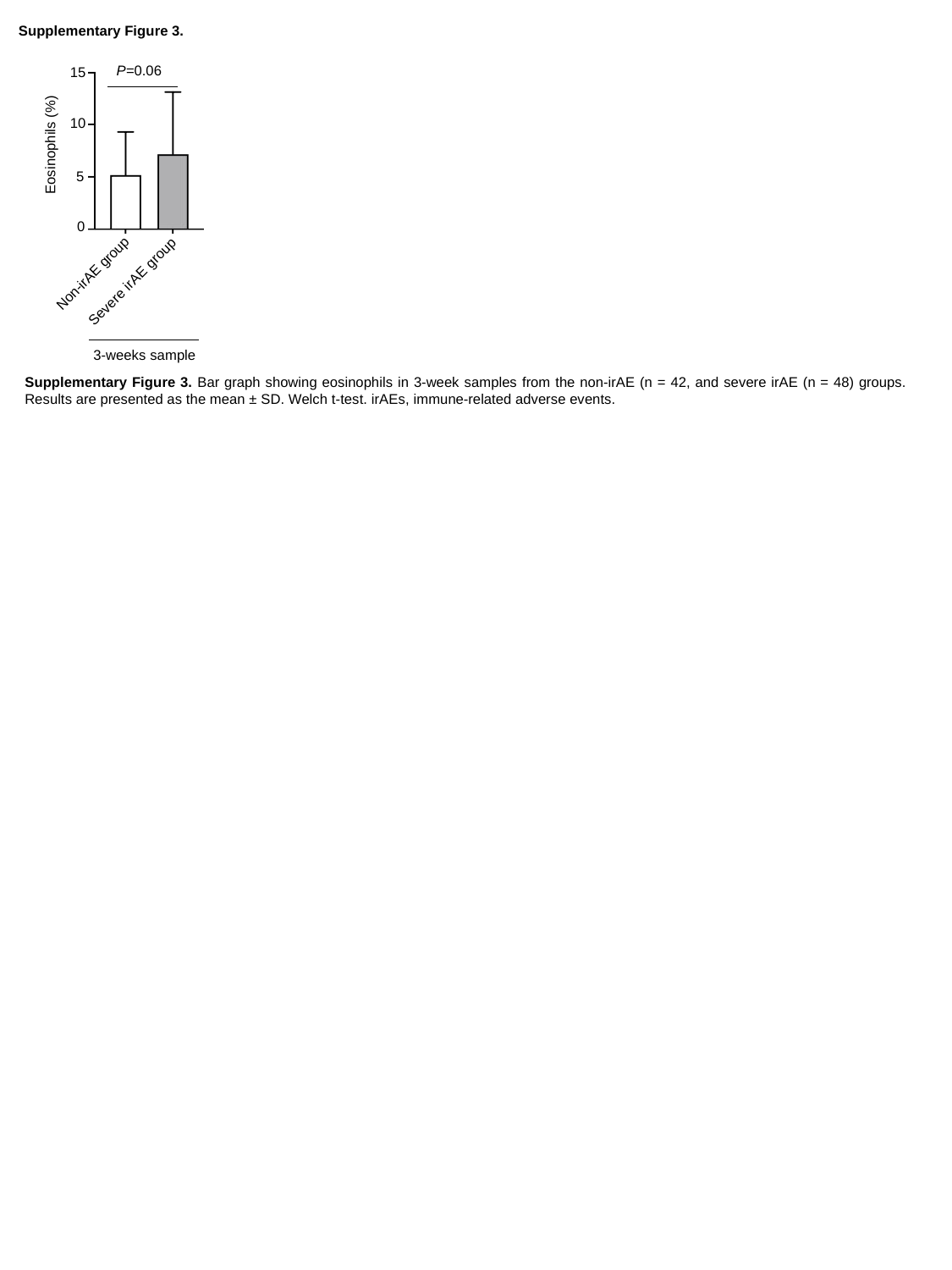

Supplementary Figure 3.
P=0.06
15
10
Eosinophils (%)
5
0
Non-irAE group
Severe irAE group
3-weeks sample
Supplementary Figure 3. Bar graph showing eosinophils in 3-week samples from the non-irAE (n = 42, and severe irAE (n = 48) groups. Results are presented as the mean ± SD. Welch t-test. irAEs, immune-related adverse events.

## Slide 4
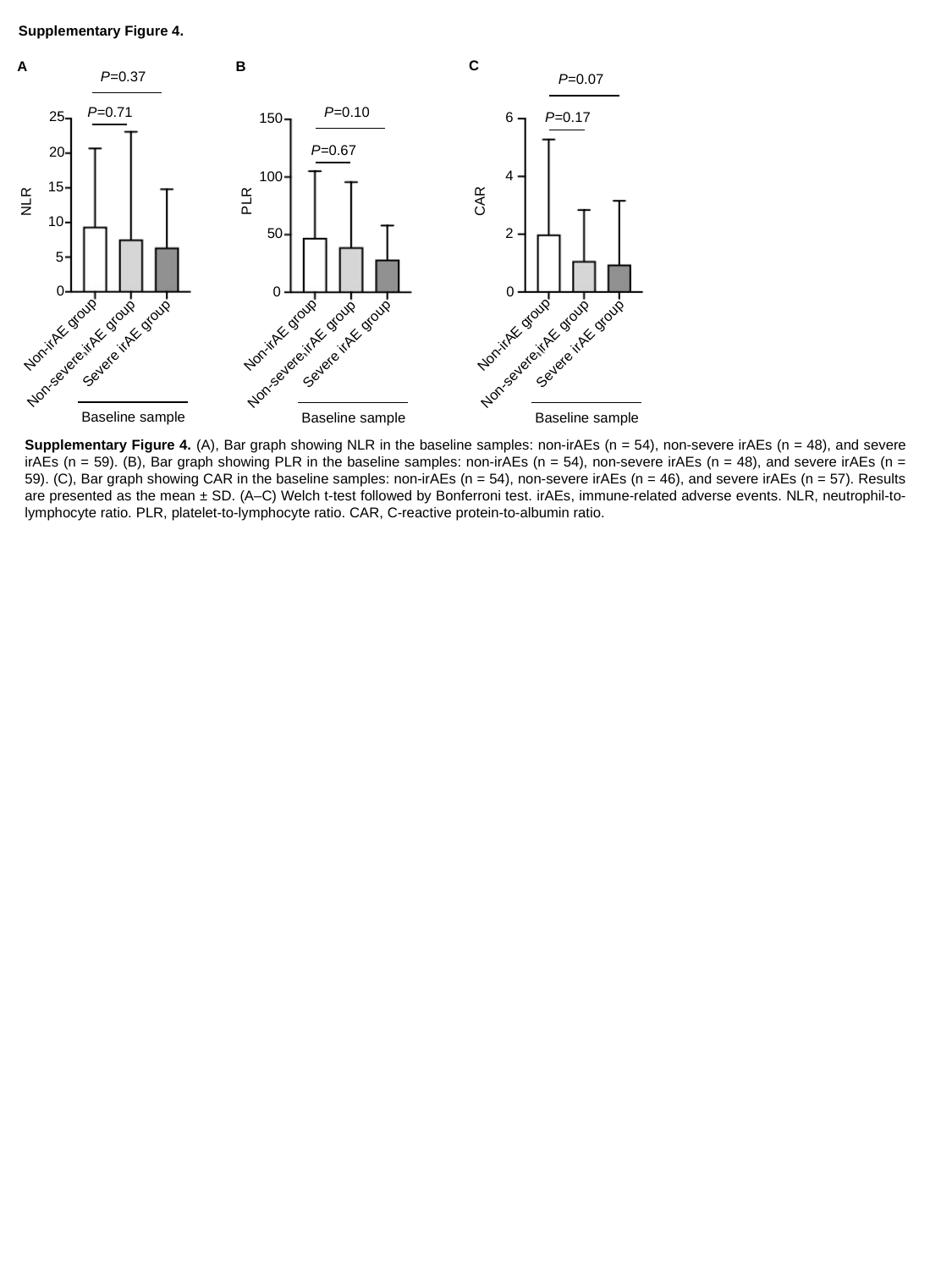

Supplementary Figure 4.
C
A
B
P=0.37
P=0.07
P=0.71
P=0.10
P=0.17
25
6
150
P=0.67
20
4
100
15
CAR
PLR
NLR
10
50
2
5
0
0
0
Non-irAE group
Non-irAE group
Non-irAE group
Non-severe irAE group
Severe irAE group
Non-severe irAE group
Severe irAE group
Non-severe irAE group
Severe irAE group
Baseline sample
Baseline sample
Baseline sample
Supplementary Figure 4. (A), Bar graph showing NLR in the baseline samples: non-irAEs (n = 54), non-severe irAEs (n = 48), and severe irAEs (n = 59). (B), Bar graph showing PLR in the baseline samples: non-irAEs (n = 54), non-severe irAEs (n = 48), and severe irAEs (n = 59). (C), Bar graph showing CAR in the baseline samples: non-irAEs (n = 54), non-severe irAEs (n = 46), and severe irAEs (n = 57). Results are presented as the mean ± SD. (A–C) Welch t-test followed by Bonferroni test. irAEs, immune-related adverse events. NLR, neutrophil-to-lymphocyte ratio. PLR, platelet-to-lymphocyte ratio. CAR, C-reactive protein-to-albumin ratio.
